# Ameliorative Effects of Osthole on Experimental Renal Fibrosis *in vivo* and *in vitro* by Inhibiting IL-11/ERK1/2 Signaling

**DOI:** 10.3389/fphar.2021.646331

**Published:** 2021-05-13

**Authors:** Fan Wu, Yan Zhao, Qingqing Shao, Ke Fang, Ruolan Dong, Shujun Jiang, Fuer Lu, Jinlong Luo, Guang Chen

**Affiliations:** ^1^ Institute of Integrated Traditional Chinese and Western Medicine, Tongji Hospital, Tongji Medical College, Huazhong University of Science and Technology, Wuhan, China; ^2^ Department of Integrated Traditional Chinese and Western Medicine, Tongji Hospital, Tongji Medical College, Huazhong University of Science and Technology, Wuhan, China; ^3^ Department of Emergency, Tongji Hospital, Tongji Medical College, Huazhong University of Science and Technology, Wuhan, China

**Keywords:** osthole, IL-11/ERK1/2 signaling, renal fibrosis, HK-2, unilateral ureteral obstruction

## Abstract

**Objectives:** Natural product, osthole, has been proven to have a protective effect on organ fibrosis, including renal fibrosis. All of these studies are mainly focused on the regulation of TGF-β/Smad signaling pathway. However, due to the pleiotropic roles of TGF-β/Smad signaling, direct TGF-β-targeted treatments are unlikely to be therapeutically feasible in clinic. Recently, the downstream IL-11/ERK1/2 signaling of TGF-β has become an attractive therapeutic target without upstream disadvantages. Based on that, this study was designed to identify the potential effects of osthole on IL-11/ERK1/2 signaling pathway in renal fibrosis.

**Methods:** The renal fibrosis model was established *in vivo* and *in vitro*, we investigated the effects of osthole on unilateral ureteral obstruction (UUO)-induced renal fibrosis and TGF-β-induced HK-2 cells. After preliminarily confirming the antifibrogenic effects of osthole and the link between its antifibrogenic effects and the inhibition of IL-11/ERK1/2 signaling, we applied a direct IL-11-induced HK-2 cells fibrosis model to further explore the inhibitory effects of osthole on IL-11/ERK1/2 signaling pathway.

**Results:** Our results confirmed that osthole can decrease the secretion of fibrosis proteins, such as α-smooth muscle actin (α-SMA), collagen I, and fibronectin, ameliorate experimental renal fibrosis *in vivo* and *in vitro*, and the effect was associated with suppressing TGF-β1/Smad signaling. More importantly, we found that IL-11/ERK1/2 signaling in UUO-induced renal fibrosis and TGF-β-induced HK-2 cell model was obviously upregulated, and osthole treatment also significantly inhibited the abnormal IL-11/ERK1/2 signaling activation. Given the direct link between TGF-β/Smad signaling and IL-11/ERK1/2 signaling pathway, we have verified that osthole has a direct inhibitory effect on IL-11/ERK1/2 signaling independent of TGF-β signaling by using an IL-11-induced HK-2 cells fibrosis model. Osthole treatment decreased the protein expression of α-SMA, collagen I and fibronectin without changing their mRNA levels in IL-11-induced HK-2 cells. Moreover, it was observed that the IL-11/ERK1/2 inhibitor, U0126, partly blocked the antifibrogenic effects of osthole.

**Conclusion:** In this study, we found that osthole has a previously unrecognized role in inhibiting IL-11/ERK1/2 signaling pathway. Our work demonstrated that the antifibrogenic effect of osthole is not only mediated by TGF-β/Smad2/3 signaling, but also directly mediated by IL-11/ERK1/2 signaling pathway independent of TGF-β1 signaling.

## Introduction

Chronic kidney disease (CKD) has become a worldwide health challenge, which brings the great concerns on public health and leads to a massive increase in healthcare burden (Levey and Coresh, 2012). CKD can be caused by various factors, such as diabetes, hypertension, nephrotic syndrome and others, and ultimately progresses to renal failure. Currently, there is still a lack of satisfactory therapeutic strategy for that in clinic. Renal fibrosis is the final common pathological feature in almost all kinds of CKDs, especially in the obstructive nephropathy, which is characterized by ECM deposition, fibroblast activation, tubular atrophy, and inflammatory cell infiltration (Boor et al., 2010; Zeisberg and Neilson, 2010). These histopathological changes of renal fibrosis directly lead to the loss of renal function and the development of end-stage renal damage (Kaissling et al., 2013). Therefore, protecting renal fibrosis is the crucial step for exploring the effective treatments for CKDs (Tampe and Zeisberg, 2014).

It is well known that transforming growth factor-β1 (TGF-β1) is the principal profibrotic factor in organ fibrosis (Schafer et al., 2017). The activation of TGF-β1/Smad signaling directly promotes the transcription of fibrogenic genes, like *α-SMA*, *collagen* and *fibronectin* (Meng et al., 2015), and induced the characteristic pathological manifestations. Targeting TGF-β1 signaling has been proven to have certain effects on organ fibrosis. However, due to the pleiotropic roles of TGF-β1/Smad signaling, its inhibition maybe be related to many side effects (Shull et al., 1992; Bierie et al., 2009). Thus, the downstream molecules of TGF-β1 could be attractive therapeutic targets without upstream disadvantages.

Interleukin-11 (IL-11) is a member of IL-6 family with distinct biological functions. IL-11 was obviously up-regulated in response to TGF-β1 stimulation, and its expression level was strongly correlated with fibroblast activation (Schafer et al., 2017). Unlike IL-6, a classic inflammatory cytokine that signals predominantly via JAK/STAT pathway, recent studies demonstrated that IL-11 causes sustained ERK1/2 activation without activation of STAT pathway (Widjaja et al., 2019). In contrast to TGF-β1, which exerts a remarked effect on the transcription of fibrogenic genes, IL-11 enhances the translation of fibrogenic genes through ERK1/2 signaling (Schafer et al., 2017; Ng et al., 2019; Corden et al., 2020), with a negligible effect on the transcriptional levels. In clinic, IL-11 was initially used to treat chemotherapy-induced thrombocytopenia due to its ability to stimulate megakaryocyte colony formation (Kawashima and Takiguchi, 1992; Isaacs et al., 1997). However, the clinical use of IL-11 brought some common side effects, include atrial arrhythmia, pulmonary congestion and others (Liu et al., 2019; Smith, 2000). These evidences suggested that IL-11 may have a pathogenic role in fibrotic cardiac disease. Meanwhile, animal studies demonstrated that the inhibition of IL-11/ERK1/2 signaling by IL-11 receptor (IL-11RA) knock out or ERK1/2 inhibitors administration apparently relieved the progression of organ fibrosis (Schafer et al., 2017; Corden et al., 2020). Taken together, these findings suggested that the regulation of IL-11/ERK1/2 axis may be a promising strategy for combating renal fibrosis.

*Cnidium monnieri* (L.) Cusson (Chinese name: She chuang zi) is an important herbal medicine that has been used to treat chronic renal diseases, female genitals, male impotence, frigidity, and skin diseases for centuries in China (Sun et al., 2020). It was documented that the biological activities of *Cnidium monnieri* (L.) Cusson are mainly mediated by its coumarin components, among which osthole has been studied extensively and is considered to play a vital role in the clinical efficacy of this herb (Li et al., 2015). So, this is why we are interested in this component. Studies have indicated that osthole possesses various pharmacological effects, such as anti-inflammatory, antioxidative, anti-tumor effects, and others (Tsai et al., 2015; Wang et al., 2015; Zhang et al., 2015). The protective effect of osthole on organ fibrosis has been studied. It was found that osthole exerted the obvious protective effects on liver, pulmonary and myocardial fibrosis (Chen et al., 2011; Hao and Liu, 2016; Liu et al., 2015). Additionally, a recent study also indicated that osthole can ameliorate renal fibrosis by suppressing TGF-β1/Smad signaling pathway and epithelial-to-mesenchymal transition (EMT) (Zhang et al., 2018). However, all of these studies mainly focus on the effects of osthole on TGF-β1/Smad signaling pathway, and few studies were conducted to investigate the further downstream mechanisms of TGF-β1 signaling by osthole treatment. Besides, no studies report the pharmacological effects of osthole on the novel therapeutic targets, IL-11/ERK1/2 axis, in renal fibrosis. Thus, this study was designed to address the pharmacological effects of osthole on renal fibrosis and figure out the underlying downstream mechanisms of osthole on TGF-β1 signaling. Our study established osthole having a previously unrecognized role in inhibiting IL-11/ERK1/2 signaling pathway, which provided a new idea for its mechanism explanation and further clinical application.

## Materials and Methods

### Antibodies and Chemicals

Collagen I and *α*-SMA antibodies were from Abcam (Cambridge, MA); *p*-Smad2/3 and Smad2/3 antibodies were obtained from Cell Signaling Technology (Beverly, MA, United States); Fibronectin and ERK1/2 antibodies were purchased from Proteintech (Wuhan, China); IL-11 and *p*-ERK1/2 antibodies were obtained from ABclonal Technology (Wuhan, China); IL-11RA antibody was from Santa Cruz Biotechnology (Santa Cruz, CA); GAPDH antibody was obtained from Wuhan Gugeshengwu Technology Co., Ltd (Wuhan, China); all secondary antibodies were purchased from Cell Signaling Technology (Beverly, MA, United States).

**FIGURE 1:**
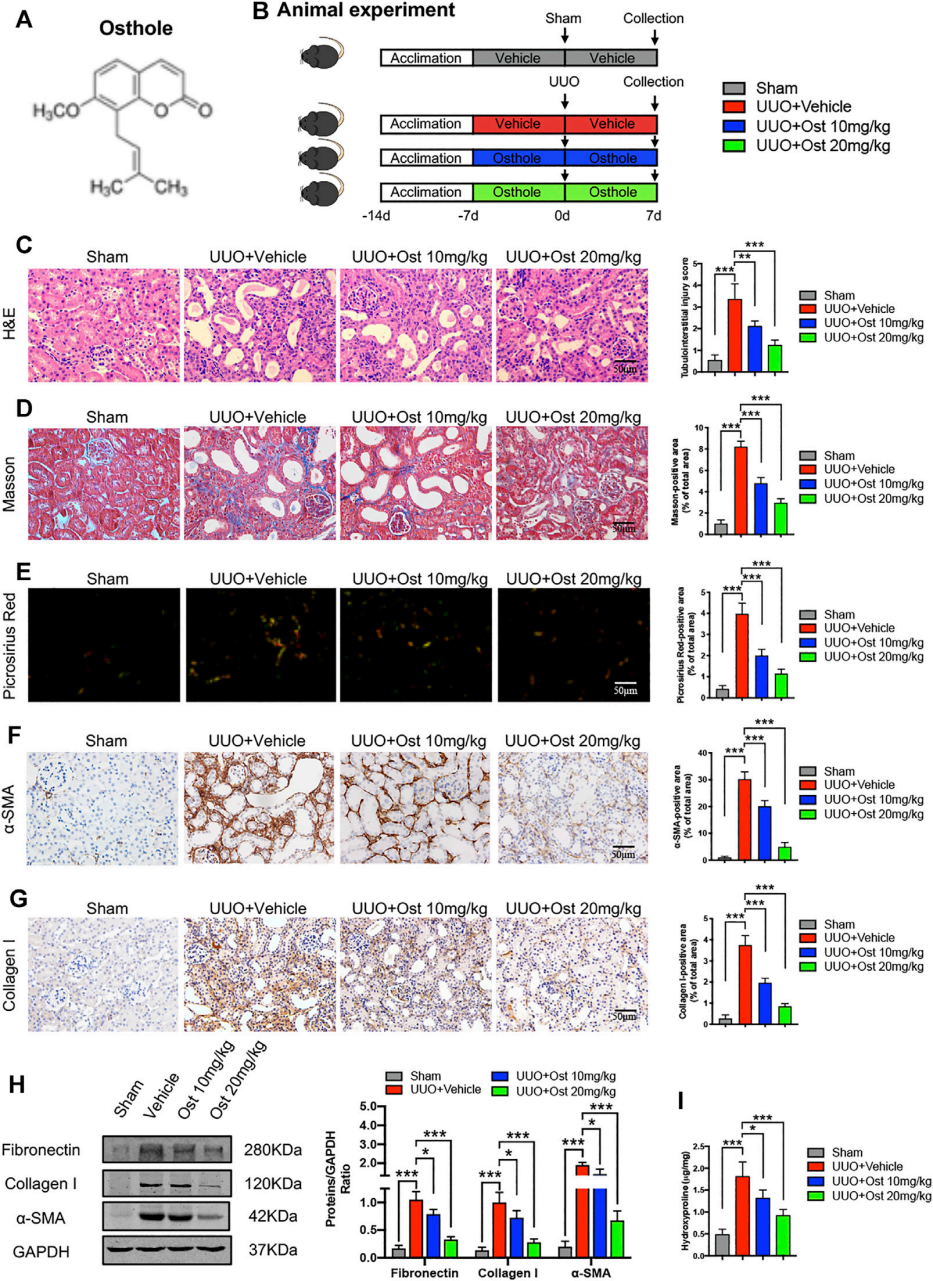
Osthole ameliorates UUO-induced renal pathological damage and renal fibrosis *in vivo.* **(A)** Chemical structure of osthole. **(B)** Animal experimental protocol of this study. **(C)** Representative kidney H&E staining of different groups; the quantification of tubulointerstitial injury score. Scale bar, 50 μm. (*n* = 6 mice/group) **(D)** Representative kidney Masson’s trichrome staining of different groups; the quantification of Masson’s trichrome staining. Scale bar, 50 μm. (*n* = 6 mice/group) **(E)** Representative kidney Picrosirius Red staining of different groups; the quantification of Picrosirius Red staining. Scale bar, 50 μm. (*n* = 6 mice/group) **(F)** Representative immunohistochemistry staining for *α*-SMA; the quantification of *α*-SMA immunohistochemistry staining. Scale bar, 50 μm. (*n* = 6 mice/group) **(G)** Representative immunohistochemistry staining for collagen I; the quantification of collagen I immunohistochemistry staining. Scale bar, 50 μm. (*n* = 6 mice/group). **(H)** Representative western blots for *α*-SMA, collagen I and fibronectin protein expressions in kidney; the quantification of *α*-SMA, collagen I and fibronectin western blots. (*n* = 6 mice/group) **(I)** Kidney hydroxyproline level of different groups. (*n* = 6 mice/group). All data are presented as means ± SD. *p < 0.05, **p < 0.01, ***p < 0.001.

Osthole (Ost, Figure 1A) was obtained from Sigma-Aldrich (St. Louis, MO); Fetal bovine serum (FBS) was purchased from Gibco (Thermo Fisher Scientific, Waltham, MA); Dulbecco’s modified Eagle medium/Ham’s F-12 medium (DMEM/F12) was purchased from Hyclone Laboratories Inc (Logan, UT, United States); Recombinant human TGF-β1 and IL-11 were obtained from Peprotech (Rocky Hill, NJ, United States); U0126 was provided by Selleck Chemicals (Shanghai, China); Trizol, Hifair^™^ II 1st Strand cDNA Synthesis Super Mix and Hieff^®^qPCR SYBR^®^Green Master Mix were purchased from Yeasen (Shanghai, China); The immunohistochemistry kit was from Wuhan Gugeshengwu Technology Co., Ltd. (Wuhan, China). All other regular reagents were obtained from Wuhan Gugeshengwu Technology Co., Ltd. unless otherwise specified.

### Animals and Treatment

The animal studies were overseen and approved by the Animal Ethics Committee of Tongji Medical College, Huazhong University of Science and Technology before and during the experiment. Thirty-two 8 week-old male C57BL/6 mice were purchased from Hubei Province Experimental Animal Research Center (SPF-grade) and housed in an environmentally controlled breeding room (temperature: 20 ± 2°C, humidity: 60 ± 5%, 12°h dark/light cycle). Water and food were given *ad libitum* throughout the study period. After one-week of adaptive feeding, the mice were randomly divided into four groups (six mice in each group): sham group, model group, 10 mg/kg osthole group and 20 mg/kg osthole group. All mice were received oral gavage of vehicle or osthole daily, and drug treatment lasted for the next 2°weeks until the end of this study. Unilateral ureteral obstruction (UUO) or sham surgery was performed as described previously at the middle of drug treatment (Cheng et al., 2010). Briefly, the mice were anesthetized with 1% pentobarbital (65 μl/10 g), the left ureter was exposed by a midline incision, and then isolated and ligated with 4–0 silk sutures. Sham-operated mice followed the same procedure but their ureters were not ligated. Finally, animals were sacrificed under anesthesia and the kidneys were harvested. The experimental protocol is illustrated in Figure 1B.

### Cell Culture and Treatment

Human proximal tubular cell line HK-2 was kindly provided by the Department of nephrology, Tongji hospital, Tongji Medical College, Huazhong University of Science and Technology. HK-2 cells were cultured in DMEM/F12 supplemented with 10% FBS, and maintained at 37°C in a humidified atmosphere of 5% CO_2_ and 95% air. The cells were synchronized by culturing in medium without FBS for 12 h before it’s used in experiments. After synchronizing, recombinant human TGF-β1 or IL-11, at a final concentration of 10 ng/ml, was added to the culture medium to induce fibrosis *in vitro.* Osthole treatment begins with the TGF-β1 induction, and lasted for an additional 24 h before harvesting. Osthole used in HK-2 cells was dissolved in 0.1% DMSO, and the equivalent amount of DMSO were applied as control.

### Cell Viability Assay

Cell viability was examined by the MTT assay according to the manufacturer’s protocol. Briefly, HK-2 cells were seeded at 1 × 10^4^ cells/well in 96-well plates. After cells adhered, the medium was replaced with DMEM/F12 containing different concentrations of osthole and maintained for another 24 h. 20°μl MTT working solution (5 mg/ml) was added to the medium. Then, the cells were incubated at 37°C for 4–6 h. The absorbance at 570 nm was measured on a Synergr2 multifunctional microplate reader (Bio-Tek, United States).

### Kidney Histopathology

For histological analysis, kidney tissues were fixed in 10% formalin, embedded in paraffin, and cut into sections (4°μm thick), which were stained with hematoxylin and eosin (H&E), Masson’s trichrome and Picrosirius Red according to the manufacturer protocol. The tubulointerstitial injury level was evaluated by grading the tubular dilatation, brush border loss, and epithelial simplification. According to the extent of cortical involvement, the degree of damage between none (normal), <25, 25–50, 50–75, and >75% were assigned as 0, 1, 2, 3, and 4 points, respectively. At least 10 randomly non-overlapping cortical fields of each section were selected and evaluated in a blinded manner. Finally, the mean score of each tissue was calculated as the final tubulointerstitial injury score. The collagen deposition was assessed by Masson’s trichrome and Picrosirius Red staining. Images from each section were acquired using an Olympus BX51 system (Olympus, Japan) and analyzed, quantified using ImageJ software (National Institutes of Health, United States).

### Immunohistochemistry Staining

For immunohistochemistry (IHC) staining, kidney sections were dewaxed by dimethylbenzene, polarized with descending concentrations of alcohol (75, 95, 100%, 5 min each), and rinsed with deionized water. After that, antigen retrieval was performed by heat-induced epitope retrieval using citrate buffer (10 mM, pH 6.0). The sections were heated to 98°C in microwave, and then maintained at a sub-boiling temperature for 10 min. Then, 3% H_2_O_2_ solution was used to block the endogenous peroxidase activity (room temperature, 30 min). The sections were blocked with 10% normal goat serum for 1 h and incubated at 4°C overnight with primary antibodies. At next day, horseradish peroxidase (HRP)-conjugated secondary antibodies were applied for 60 min at room temperature, and these sections were visualized by DAB and counterstained with hematoxylin. Images from each section were captured using an Olympus BX51 system (Olympus, Japan) and analyzed by ImageJ software.

### Immunofluorescence Staining

Cultured cells were fixed in 10% formalin for 30 min and blocked with 10% normal goat serum for 1 h. After that, the cells were incubated with primary antibodies at 4°C overnight, followed by staining with Alexa Flour 488 or CY3-conjugated secondary antibody. The nuclei were stained using DAPI or PI solution and all operations were carried out under the light-protected conditions after incubating the secondary antibody. The images were taken by Olympus BX51 fluorescent microscope and analyzed, quantified using ImageJ software.

### Western Blot Analysis

Total proteins were extracted from kidney tissues and HK-2 cells using standard protocols, and then protein concentrations were quantified by the bicinchoninic acid (BCA) protein assay kit. An equal amounts of protein extracts were loaded on SDS-PAGE (80 V, 0.5 h and then 120 V, 1 h) and electrotransferred to a 0.45 μm nitrocellulose membrane (280 mA, 1 kDa/min). The membranes were blocked with 5% nonfat milk for 1 h at room temperature and incubated with primary antibodies overnight at 4°C. On the next day, fluorescence-conjugated secondary antibodies were applied to the membranes for 1 h at room temperature. The membranes were visualized with Odyssey Infrared Imaging (LI-COR Biosciences, United States). Target proteins were normalized to GAPDH and quantified by ImageJ software.

### Real-Time Quantitative Polymerase Chain Reaction

Total RNA was extracted from kidney tissues and HK-2 cells using Trizol reagent according to the standard protocol. After reverse-transcription, RT-qPCR was performed on LightCycler^®^96 system (Roche Diagnostics, Mannheim, Germany). The mRNA level of target genes was normalized and analyzed by the 2^−ΔΔCT^ method. Sequences of the primers used in this study are listed in Table 1.

**TABLE 1:** Primers used for RT-qPCR.

Gene	Forward (5′-3′)	Reverse (5′-3′)
H-GAPDH	GGA​AGC​TTG​TCA​TCA​ATG​GAA​ATC	TGA​TGA​CCC​TTT​TGG​CTC​CC
H-α-SMA	CCAGCCATCCTTCATCGG	GAT​CCA​GAC​AGA​GTA​TTT​GCG​C
H-collagen I	TGG​CAA​AGA​TGG​ACT​CAA​CG	TCA​CGG​TCA​CGA​ACC​ACA​TT
H-fibronectin	GGA​GAG​TGG​AAG​TGT​GAG​AGG​C	TCC​ATT​TGA​GTT​GCC​ACC​GT
H-IL-11	CAC​AAC​CTG​GAT​TCC​CTG​CC	CCC​AGT​CAA​GTG​TCA​GGT​GCA
H-IL-11RA	GGG​ACC​ATA​CCA​AAG​GAG​ATA​CC	TCC​CAA​AGA​CGC​CAG​CAC​A
M-IL-11	CTG​GGA​CAT​TGG​GAT​CTT​TGC	TAC​ATG​CCG​GAG​GTA​GGA​CA
M-IL-11RA	GGAGGCCTCCAGAGGGT	GGG​TCC​TCC​AGG​GGT​CCA​GTA​TCC
M-GAPDH	CCT​CGT​CCC​GTA​GAC​AAA​ATG	TGA​GGT​CAA​TGA​AGG​GGT​CGT

### Statistical Analysis

All data are presented as means ± SD. Statistical analyses were performed followed this rule: Firstly, the normality of data is tested by Shapiro-Wilk test; Secondly, data fits the normal distribution are tested for homogeneity of variance via one-way ANOVA; Finally, Tukey’s multiple comparisons test can be performed to compare multiple groups only if there is no significant variance inhomogeneity between groups. Mann-Whitney *U*-test should be used as an alternative post hoc test when the data show non-normality or variance inhomogeneity. Statistics were analyzed using the GraphPad Prism 8 software, and *p* < 0.05 was considered as statistically significant.

## Results

### Osthole Ameliorates Unilateral Ureteral Obstruction-Induced Renal Pathological Damage and Renal Fibrosis *in vivo*

To verify the protective effects of osthole on renal fibrosis, UUO-induced *in vivo* renal fibrosis model was established in our study. As shown in Figure 1C, compared to sham group, there was an obvious tubulointerstitial injury including tubular dilatation, epithelial atrophy and interstitial cell infiltration. Osthole administration significantly ameliorated UUO-induced renal pathological damage (Figure 1C). Masson’s trichrome and Picrosirius Red staining were used to intuitively reflect the fibrosis levels between different groups. Both of these results indicated that model group has remarkable collagen deposition in interstitial area, while in the sham group, almost no significant fibrosis is observed. After osthole treatment, UUO-induced renal fibrosis was significantly ameliorated (Figures 1D,E). In addition, we further detected the expression of fibrosis markers (like *α*-SMA, collagen I and fibronectin) by IHC and western blot. As expected, osthole administration can significantly inhibit the secretion of *α*-SMA, collagen I and fibronectin, which directly leads to the improvement of renal fibrosis (Figures 1F–H). Hydroxyproline, a peculiar amino acid in collagen tissue, is considered as a sensitive biomarker for fibrosis level. Consistently, compared to control group, osthole treatment apparently reversed the elevated hydroxyproline level (Figure 1I). All together, these data indicated that osthole ameliorates UUO-induced renal pathological damage and renal fibrosis *in vivo*.

### Osthole Ameliorates Transforming Growth Factor-β-Induced HK-2 Cells Fibrosis *in vitro*

**FIGURE 2:**
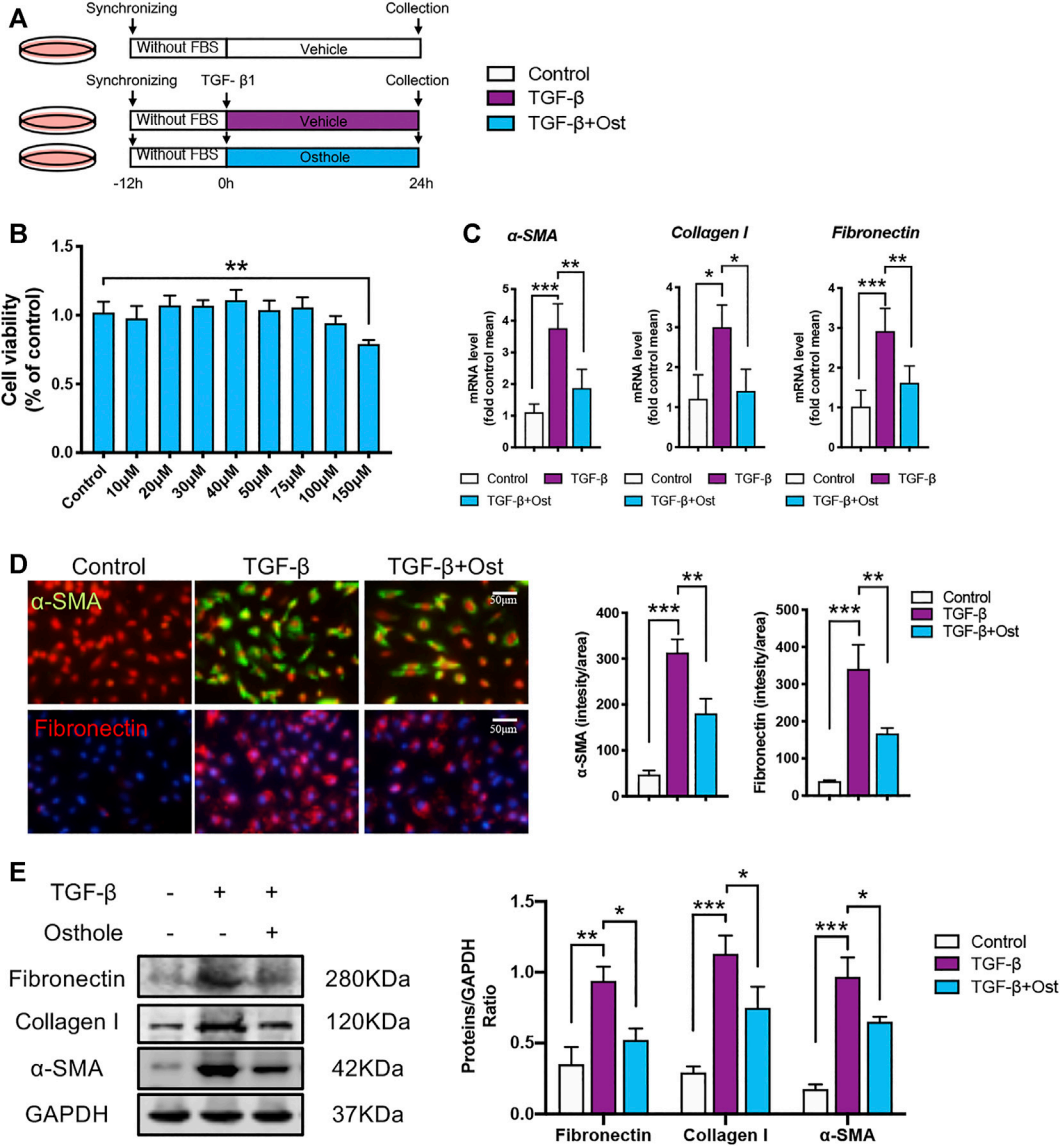
Osthole ameliorates TGF-β-induced HK-2 cells fibrosis *in vitro.* **(A)** *in vitro* experimental protocol of this part. **(B)** MTT assay was applied to determine the concentrations of osthole. (*n* = 4 repeats) **(C)** The mRNA levels of *α*-SMA, collagen I and fibronectin in HK-2 cells of different groups. (*n* = 4 repeats) **(D)** Representative immunofluorescence staining for *α*-SMA and fibronectin; the quantification of α-SMA and fibronectin immunofluorescence staining. Scale bar, 50 μm. (*n* = 4 repeats) **(E)** Representative western blots for *α*-SMA, collagen I and fibronectin protein expressions in HK-2 cells; the quantification of *α*-SMA, collagen I and fibronectin western blots. (*n* = 4 repeats). All data are presented as means ± SD. *p <0.05, **p < 0.01, ***p < 0.001.

After confirming the protective effects of osthole on renal fibrosis *in vivo*, we further explored the ameliorative effects of osthole on renal fibrosis *in vitro* as described previously (Figure 2A). The maximum nontoxic concentration of osthole used *in vitro* was 100 μM (Figure 2B). Firstly, the mRNA levels of *α*-SMA, collagen I and fibronectin were determined by RT-qPCR. Compared to control group, TGF-β induction led to the obvious increases in these fibrosis proteins transcription, while osthole treatment showed an inhibitory effect on mRNA expression levels of fibrosis proteins (Figure 2C). Then, we performed Immunofluorescence staining on *α*-SMA and fibronectin, the results were similar to that of RT-qPCR. Treatment of osthole significantly reduced the excessive expression of *α*-SMA and fibronectin (Figure 2D). In addition, the protein expression levels of *α*-SMA, collagen I and fibronectin were also verified by western blot. Figure 2E showed that TGF-β induced an obvious fibrosis phenotype in HK-2 cells compared with control group, while osthole administration led to a significant reduction in fibrosis proteins expression (Figure 2E). Our results demonstrated that osthole can also exert the ameliorative effects on TGF-β-induced HK-2 cells fibrosis *in vitro.*

### Osthole Inhibits IL-11/ERK1/2 Signaling Pathway in Experimental Renal Fibrosis Model *in vivo* and *in vitro*.

Since the ameliorative effects of osthole on experimental renal fibrosis *in vivo* and *in vitro* have been confirmed, we planned to evaluate the underlying mechanism on the antifibrogenic effects of osthole. Recent study has confirmed that osthole inhibits classic TGF-β/Smad signaling in renal fibrosis, which was also verified in our study. Both in *vivo* and *in vitro* models, osthole treatment significantly inhibited the activation of Smad2/3 (Supplementary Figure S1). As mentioned above, few studies were conducted to investigate the further downstream mechanisms of TGF-β1 signaling by osthole treatment. Considering the critical role of IL-11, an important downstream effector of TGF-β1 signaling, in the progression of renal fibrosis, thus the key molecules of IL-11/ERK1/2 axis were examined.

**FIGURE 3:**
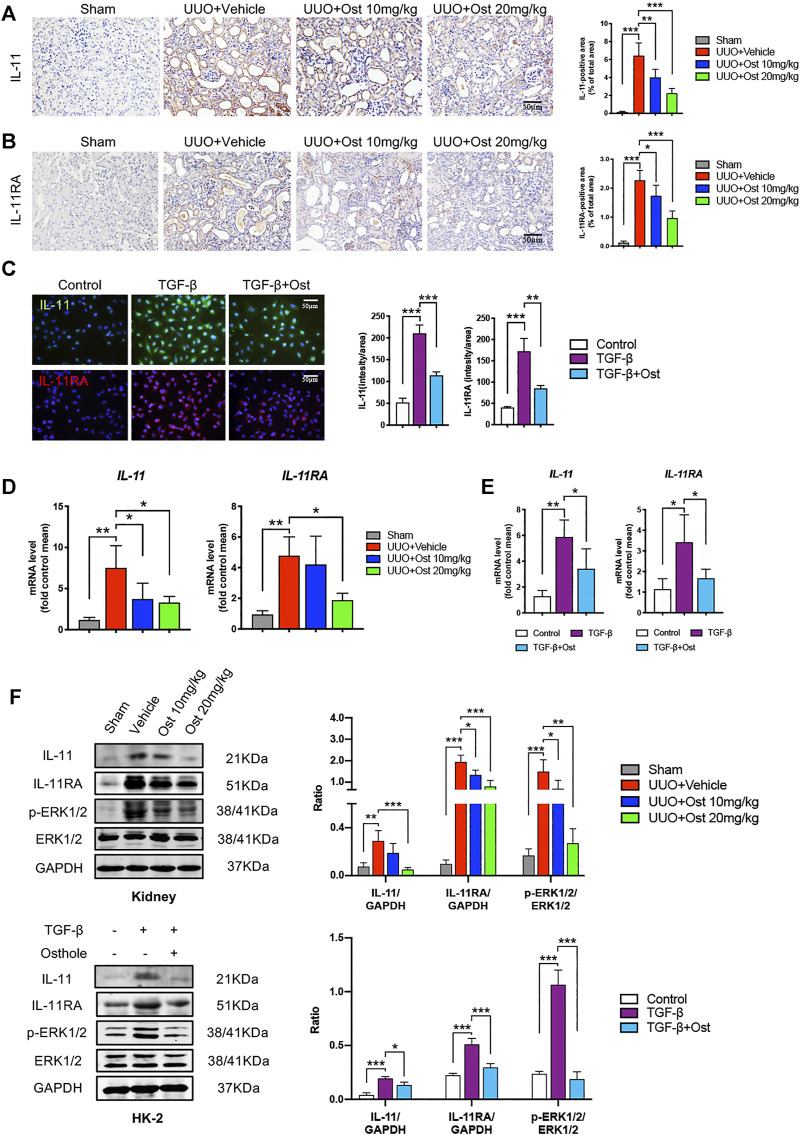
Osthole inhibits IL-11/ERK1/2 signaling pathway in experimental renal fibrosis model *in vivo* and *in vitro.* **(A)** Representative immunohistochemistry staining for IL-11; the quantification of IL-11 immunohistochemistry staining. Scale bar, 50 μm. (*n* = 6 mice/group) **(B)** Representative immunohistochemistry staining for IL-11RA; the quantification of IL-11RA immunohistochemistry staining. Scale bar, 50 μm. (*n* = 6 mice/group) **(C)** Representative immunofluorescence staining for IL-11 and IL-11RA; the quantification of IL-11 and IL-11RA immunofluorescence staining. Scale bar, 50 μm. (*n* = 4 repeats) **(D)** The mRNA levels of IL-11 and IL-11RA in kidney of different groups. (*n* = 6 mice/group) **(E)** The mRNA levels of IL-11 and IL-11RA in HK-2 cells of different groups. (*n* = 4 repeats) **(F)** Representative western blots for IL-11, IL-11RA, *p*-ERK1/2 and ERK1/2 protein expressions in kidney and HK-2 cells; the quantification of IL-11, IL-11RA, *p*-ERK1/2 and ERK1/2 western blots. (*n* = 6 mice/group or 4 repeats *in vitro*). All data are presented as means ± SD. *p <0.05, **p < 0.01, ***p < 0.001.

Firstly, in order to figure out whether IL-11 signaling was upregulated in renal fibrosis and its changing trend under osthole treatment. The protein expressions of IL-11 and IL-11RA were determined *in vivo* and *in vitro.* As shown in Figures 3A–C, in UUO-induced *in vivo* renal fibrosis, the IL-11 and IL-11RA expressions of model group were largely increased compared with sham group. In TGF-β-induced *in vitro* fibrosis model, the IL-11 and IL-11RA expressions were also upregulated compared with control group. Osthole treatment significantly reduced the protein expressions of IL-11 and IL-11RA *in vivo* and *in vitro* (Figures 3A–C). These results suggested the antifibrogenic effects of osthole may be associated with its inhibition on IL-11 signaling. These results were also confirmed by RT-qPCR (Figures 3D,E). Next, we further assessed the important downstream signal molecule of IL-11: ERK1/2. Western blot analysis of IL-11/ERK1/2 axis both *in vivo* and *in vitro* was conducted to explore the underlying mechanisms. It was observed that IL-11/ERK1/2 signaling in renal fibrosis model was significantly upregulated, which was consistent with reported previously (Schafer et al., 2017). Osthole treatment significantly decreased the expressions of IL-11, IL-11RA, and p-ERK1/2, and inhibited the abnormal IL-11/ERK1/2 signaling activation. These results preliminarily verified our hypothesis and suggested that the antifibrogenic effects of osthole may not only be associated with TGF-β/Smad2/3 signaling, but may also be mediated by IL-11/ERK1/2 signaling pathway.

### The Antifibrogenic Effects of Osthole can be Mediated by IL-11/ERK1/2 Signaling Pathway

However, mechanically, TGF-β1 signaling activation can lead to an obvious improvement in the transcription of fibrogenic genes, and our research object IL-11 is also one of these target genes. This phenomenon was also observed in our study, TGF-β stimulation led to the obvious increases of IL-11 and IL-11RA mRNA expression levels (Figure 3E). It can be expected that the inhibition of TGF-β1 signaling can reduce IL-11 expression, thereby inhibiting IL-11/ERK1/2 signaling pathway. Thus, there is a question whether the inhibition of osthole on IL-11/ERK1/2 signaling pathway is fully attributed to its inhibitory effect on upstream TGF-β1 signaling, or whether there is another mechanism that mediates the inhibition of osthole on IL-11/ERK1/2 signaling pathway.

**FIGURE 4:**
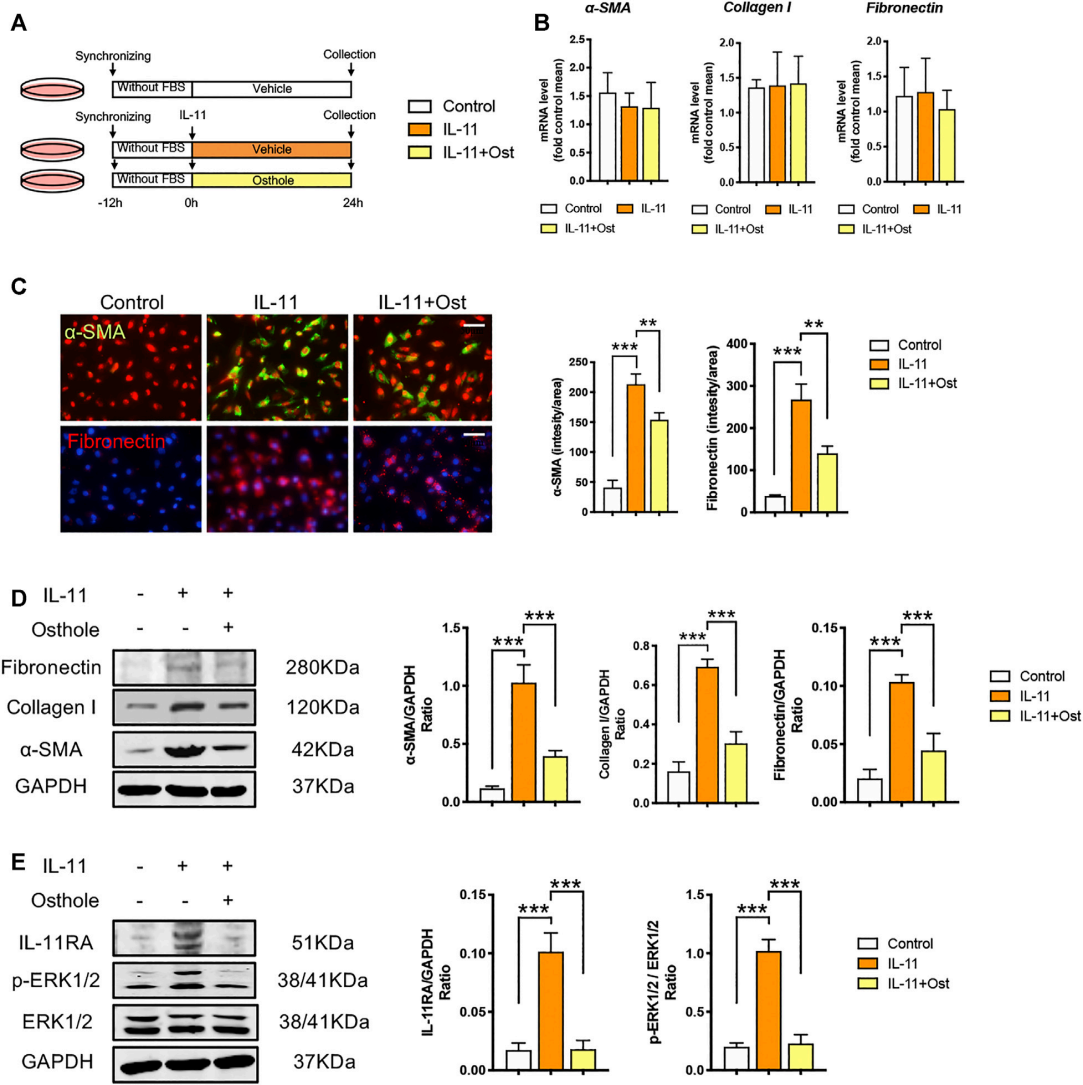
The antifibrogenic effects of osthole can be mediated by IL-11/ERK1/2 signaling pathway. **(A)** *in vitro* experimental protocol of this part. **(B)** The mRNA levels of *α*-SMA, collagen I and fibronectin in HK-2 cells of different groups. (*n* = 4 repeats) **(C)** Representative immunofluorescence staining for *α*-SMA and fibronectin; the quantification of *α*-SMA and fibronectin immunofluorescence staining. Scale bar, 50 μm. (*n* = 4 repeats) **(D)** Representative western blots for *α*-SMA, collagen I and fibronectin protein expressions in HK-2 cells; the quantification of *α*-SMA, collagen I and fibronectin western blots. (*n* = 4 repeats) **(E)** Representative western blots for IL-11RA, *p*-ERK1/2 and ERK1/2 protein expressions in HK-2 cells; the quantification of IL-11RA, *p*-ERK1/2, and ERK1/2 western blots. (*n* = 4 repeats). All data are presented as means ± SD. *p <0.05, **p < 0.01, ***p < 0.001.

Therefore, to eliminate the influence of upstream TGF-β1 signaling on mechanism studies, we applied IL-11-induced HK-2 cells model to further explore the mechanisms involved the inhibitory effects of osthole on IL-11/ERK1/2 signaling pathway (Figure 4A). Previous studies revealed that the activation of IL-11/ERK1/2 signaling mainly promotes the translation of fibrogenic genes without changes the transcriptional levels (Corden et al., 2020). Consistently, in our study, there was no statistically significance between the mRNA levels of *α*-SMA, collagen I and fibronectin in different groups under IL-11 stimulation (Figure 4B). But compared with control group, the protein expressions of *α*-SMA and fibronectin were still obviously induced by IL-11 stimulation, and osthole treatment still significantly inhibited the secretion of these fibrosis proteins (Figures 4C,D). These data showed that the activation of IL-11 signaling can induce fibrosis phenotype, and this effect is independent of TGF-β1 signaling, because the latter can obviously promote fibrosis proteins transcription. Meanwhile, we also detected the protein expressions of IL-11/ERK1/2 axis. As expected, it was found that IL-11/ERK1/2 signaling was obviously upregulated under IL-11 stimulation, while osthole administration significantly recovered this trend (Figure 4E). Taken together, our data confirmed the effects of IL-11/ERK1/2 axis on the translation of fibrogenic genes and demonstrated that the antifibrogenic effects of osthole can also be mediated by IL-11/ERK1/2 signaling pathway independent of TGF-β1 signaling.

### Interleukin-11/ERK1/2 Inhibition Partly Blocks the Antifibrogenic Effects of Osthole

**FIGURE 5:**
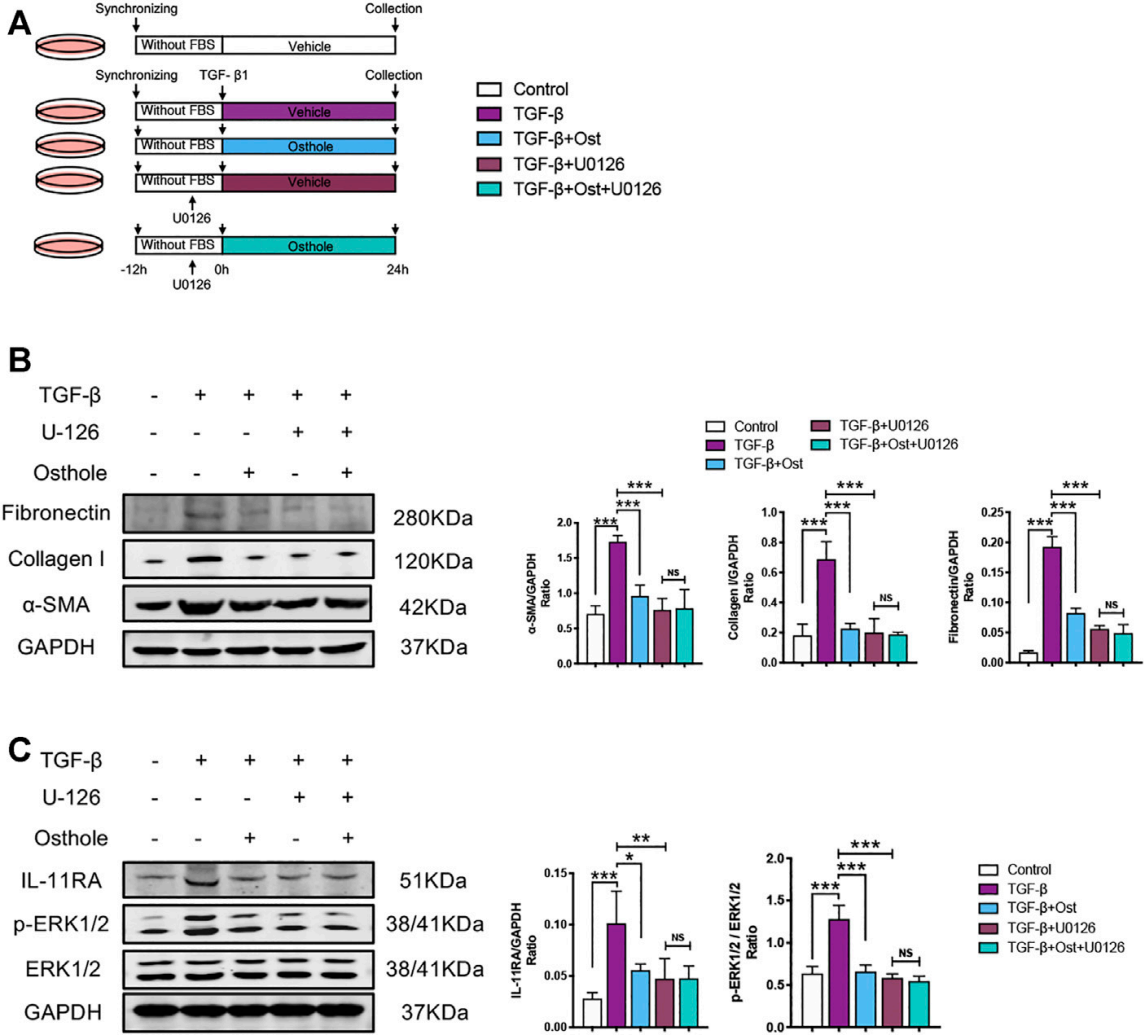
IL-11/ERK1/2 inhibition partly blocks the antifibrogenic effects of osthole. **(A)** *in vitro* experimental protocol of this part. **(B)** Representative western blots for *α*-SMA, collagen I and fibronectin protein expressions in HK-2 cells; the quantification of *α*-SMA, collagen I and fibronectin western blots. (*n* = 4 repeats) **(C)** Representative western blots for IL-11RA, *p*-ERK1/2 and ERK1/2 protein expressions in HK-2 cells; the quantification of IL-11RA, *p*-ERK1/2 and ERK1/2 western blots. (*n* = 4 repeats). All data are presented as means ± SD. *p <0.05, **p < 0.01, ***p < 0.001.

In order to further verify the role of IL-11/ERK1/2 signaling on the antifibrogenic effects of osthole, we conducted an IL-11/ERK1/2 signaling inhibition study. An ERK1/2 inhibitor, U0126, was used to inhibit IL-11/ERK1/2 signaling transduction in TGF-β-induced HK-2 cells fibrosis, while we can assess the role of IL-11/ERK1/2 signaling on the antifibrogenic effects of osthole (Figure 5A).

Compared with model group, both osthole and U0126 treatment group all significantly decreased the TGF-β-induced increases in *α*-SMA, collagen I and fibronectin protein expressions. Under U0126 application, osthole administration did not further reduce the expressions of these fibrosis proteins (Figure 5B). Similarly, the changes of IL-11/ERK1/2 signaling molecules were also consistent with above trend. IL-11/ERK1/2 signaling pathway was obviously inhibited both in osthole and U0126 treatment group, and osthole treatment did not bring another reduction in the expressions of IL-11RA and *p*-ERK1/2 when compared U0126 alone treatment group with U0126 plus osthole treatment group (Figure 5C). These results demonstrated that the inhibition of IL-11/ERK1/2 axis can partly block the antifibrogenic effects of osthole, which supported our above conclusion.

## Discussion

Renal fibrosis represents the common final pathway of nearly all chronic and progressive nephropathies, which showed a rapid increase in prevalence due to the sustained growth in CKDs, regardless of causes (Jha et al., 2013). The initial deposition of fibrotic matrix may occur in the tissue repair process after injury, and it could be resorbed when physiologic repairing can compensate for damaged tissue. Otherwise, in the present of chronic injury, fibrotic matrix deposition continues, eventually leads to organ architecture disruption, blood supply reduction and organ function damage (Wynn, 2008). Collectively, renal fibrosis and CKDs affect half of adults above age 70 and 10% of the world’s population (Humphreys, 2018). Although the therapeutic strategies for end-stage renal diseases have made great progress in the past few decades, but the outcome of renal fibrosis remains poor (Garcia et al., 2012). Therefore, there is an urgent need to seek alternative treatments for that.

Many of the pathophysiologic features underlying renal fibrosis are similar with other fibrotic diseases, like cirrhosis, intestinal fibrosis, cardiomyopathies, and idiopathic pulmonary fibrosis (Wynn, 2011; Ke and Chen, 2012; Weber et al., 2013; Wu et al., 2020). Among that, TGF-β signaling is recognized as the central mediator in renal fibrosis (Meng et al., 2015). A number of studies have demonstrated that TGF-β levels are significantly elevated in patients with various renal diseases, which is strongly correlated with the degree of renal fibrosis (Yamamoto et al., 1996; Murakami et al., 1997). Meanwhile, it was well documented that inhibiting TGF-β with neutralizing antibody, antisense oligonucleotides, inhibitors, or genetic deletion of receptors can attenuate renal fibrosis *in vivo* and *in vitro*, whereas the overexpression of TGF-β1 leads to renal fibrosis (Kopp et al., 1996; Border and Noble, 1998; Moon et al., 2006; Petersen et al., 2008; Meng et al., 2012). Mechanically, TGF-β signals through both canonical and non-canonical pathways, in which TGF-β canonical signaling via Smads family plays a major role in the development of renal fibrosis (Meng et al., 2016). Studies revealed that TGF-β signaling mediates renal fibrosis may mainly through followed pathways: 1) TGF-β directly promotes the transcription of fibrogenic genes via Smad2/3-dependent or independent manners; 2) TGF-β inhibits the degradation of ECM by suppressing matrix metalloproteinases (MMPs) and inducing natural inhibitors of MMPs; 3) TGF-β enhances the transdifferentiation of other cell types to increase myofibroblasts pool, such as epithelial-to-mesenchymal transition (EMT) and endothelial-to-mesenchymal transition (EndoMT); 4) TGF-β induces mesangial cells proliferation and loss of tubular epithelial cells, podocytes and endothelial cells (Lopez-Hernandez and Lopez-Novoa, 2012; Meng et al., 2015; Ma and Meng, 2019).

The inhibitory effects of osthole on TGF-β/Smad signaling has been well identified (Chen et al., 2011; Hao and Liu, 2016; Liu et al., 2017; Liu et al., 2015; Zhang et al., 2018). Actually, this point was also confirmed in our study, osthole treatment can significantly reduce the mRNA and protein levels of *α*-SMA, collagen I and fibronectin, inhibit TGF-β-induced HK-2 cells fibrosis *in vitro* (Figure 2). Western blot analysis of *p*-Smad2/3 and Smad2/3 showed that osthole obviously inhibited the TGF-β/Smad2/3 signaling transduction. Based on these results, we can draw a conclusion that the antifibrogenic effects of osthole are related to its inhibitory effects on TGF-β/Smad2/3 signaling. However, we must recognize that TGF-β signaling not only plays a crucial role in fibrosis, but also exerts multiple important biological effects. Thus, in clinic, direct TGF-β-targeted treatments are unlikely to be therapeutically feasible due to the involvement of TGF-β signaling in other systems (Chen et al., 2018). Increasing evidences suggested that the downstream molecules of TGF-β signaling may be attractive therapeutic targets in the treatment of fibrotic diseases (Schafer et al., 2017; Ng et al., 2019). Therefore, exploring the potential effective drugs from the downstream of TGF-β signaling will bring more meaningful value. That’s also the reason why we conducted this study.

As mentioned above, activated TGF-β signaling largely induces the transcription of fibrogenic genes, and IL-11 is one of these target genes. It was reported that IL-11 level is associated with renal injury in diverse animal models and humans (Mitazaki et al., 2009; Grgic et al., 2014; Xu et al., 2014; Menendez-Castro et al., 2020). Except mild craniosynostosis, delayed tooth eruption and variable joint laxity, humans with null mutations in IL-11RA have no other major abnormalities (Nieminen et al., 2011; Keupp et al., 2013; Brischoux-Boucher et al., 2018). IL-11RA^−/−^ mice having slight developmental abnormalities in skeletal system but being otherwise healthy with a normal life span (Nandurkar et al., 1997). Thus, IL-11 signaling may be a promising target downstream of TGF-β. As a member of IL-6 family, IL-11 is a less frequently studied cytokine which has only recently been shown to be an important downstream regulator of TGF-β signaling in organ fibrosis (Schafer et al., 2017). IL-11 signals via combining IL-6 to form a heterodimer complex, which leads to a belief that the biological activities of these cytokines are partly overlapping. However, studies demonstrated that IL-11 causes ERK1/2 activation instead of JAK/STAT pathway (the classic target of IL-6) in organ fibrosis. Unlike TGF-β signaling, IL-11/ERK1/2 axis promotes the expressions of fibrosis proteins mainly by enhances the mRNA translation, and exerts a negligible effect on the transcriptional levels of fibrogenic genes. This effect seems to be dependent on the downstream targets of *p*-ERK1/2, such as eurkaryotic translation initiation factor 4E (eIF4E) and 40S ribosomal protein S6 kinase (RSK), which are closely associated with the activation of protein translation (Schafer et al., 2017; Corden et al., 2020). Additionally, another recent study indicated that the IL-11-induced protein translation may be mediated by the activation of glutamyl-prolyl-tRNA synthetase (EPRS) (Wu et al., 2020). Regardless of how the activated IL-11/ERK1/2 axis promotes fibrosis protein translation, which was consistent with our expectations. IL-11 stimulation increased the protein levels of fibrosis proteins without changing their mRNA levels (Figure 4). In IL-11-induced HK-2 cells fibrosis model, osthole inhibited the protein translation of fibrosis proteins by suppressing IL-11/ERK1/2 pathway. Our data introduced osthole possessing a previously unrecognized role in inhibiting IL-11/ERK1/2 signaling pathway, which provided a new idea for its antifibrogenic effects and supported its further clinical application. In a word, our work demonstrated that the antifibrogenic effect of osthole is not only mediated by TGF-β/Smad2/3 signaling, but also directly mediated by IL-11/ERK1/2 signaling pathway.

Of course, there are still some limitations in our study. 1) Given the direct link between TGF-β/Smad2/3 signaling and IL-11/ERK1/2 signaling pathway, a genetically engineered mouse model may provide more evidences; 2) Theoretically, we confirmed the effects of IL-11/ERK1/2 signaling induction/inhibition on fibrosis model *in vitro*, but the *in vivo* experiments on inhibiting IL-11/ERK1/2 signaling are still lacking; 3) Although, the final effects of activated IL-11/ERK1/2 axis on fibrosis proteins translation and osthole’s inhibitory activities on that have been well identified in our work. How osthole inhibits the translation of fibrosis proteins via IL-11/ERK1/2 signaling is still unclear, and more research is needed in this field.

## Conclusion

**FIGURE 6:**
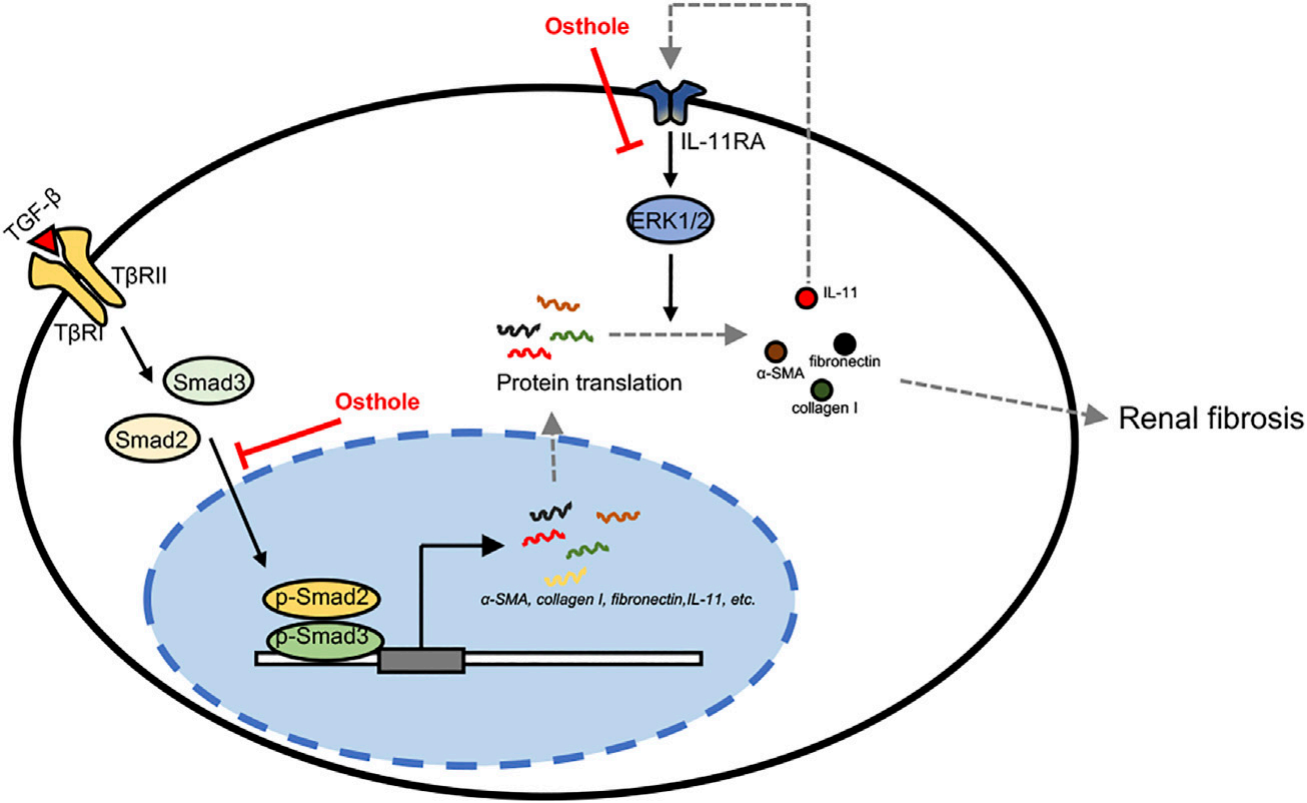
The putative mechanism of osthole’s ameliorative effects on renal fibrosis. In the progression of renal fibrosis, activated TGF-β signaling promotes the transcription of fibrogenic genes, like *α-SMA*, *collagen I, fibronectin, IL-11, etc.,* by transcription factor Smad2/3 nuclear translocation. Meanwhile, the increased IL-11 may also enhance fibrosis proteins translation via activating IL-11/ERK1/2 signaling pathway. These processes eventually contribute to ECM deposition and fibrosis. In this study, we found that osthole has a previously unrecognized role in inhibiting IL-11/ERK1/2 signaling pathway. On the one hand, osthole can inhibit the activation of TGF-β/Smad2/3 signaling, then reducing the transcription of fibrogenic genes, and thereby ameliorating fibrosis. In this process, IL-11/ERK1/2 signaling pathway is also inhibited due to IL-11 is one of the target genes of TGF-β/Smad2/3 signaling. On the other hand, osthole can directly inhibit IL-11/ERK1/2 signaling pathway independent of TGF-β1 signaling, then decreasing the translation of fibrosis proteins, finally ameliorating fibrosis. In summary, our work demonstrated that the antifibrogenic effect of osthole is not only mediated by TGF-β/Smad2/3 signaling, but also directly mediated by IL-11/ERK1/2 signaling pathway independent of TGF-β1 signaling.

In this study, we found that osthole has a previously unrecognized role in inhibiting IL-11/ERK1/2 signaling pathway. Osthole can inhibit the translation of fibrosis proteins by suppressing activated IL-11/ERK1/2 signaling in renal fibrosis, eventually contributing to the amelioration of fibrosis. In summary, our work demonstrated that the antifibrogenic effect of osthole is not only mediated by TGF-β/Smad2/3 signaling, but also directly mediated by IL-11/ERK1/2 signaling pathway independent of TGF-β1 signaling (Figure 6).

## Data Availability

The raw data supporting the conclusions of this article will be made available by the authors, without undue reservation.

## Abbreviations

BCA, bicinchoninic acid; CKD, chronic kidney disease; DMEM/F12, dulbecco’s modified Eagle medium/Ham’s F-12 medium; ECM, extracellular matrix; eIF4E, eurkaryotic translation initiation factor 4E; EMT, epithelial-to-mesenchymal transition; EndoMT, endothelial-to-mesenchymal transition; EPRS, glutamyl-prolyl-tRNA synthetase. FBS, fetal bovine serum; HRP, horseradish peroxidase; IHC, immunohistochemistry; MMP, matrix metalloproteinases; OST, osthole; IL-11, interleukin-11; RSK, 40S ribosomal protein S6 kinase; RT-qPCR, real-time quantitative polymerase chain reaction; TGF-β, transforming growth factor-β; UUO, unilateral ureteral obstruction; α-SMA, α-smooth muscle actin
