# Supplementary material for: Ameliorative Effects of Osthole on Experimental Renal Fibrosis in vivo and in vitro by Inhibiting IL-11/ERK1/2 Signaling
Source: Front Pharmacol. 2021 May 13;12:646331. doi: 10.3389/fphar.2021.646331 (PMC8155534; doi:10.3389/fphar.2021.646331)
Supplement: Supplementary file 1 [file Image1.pdf]

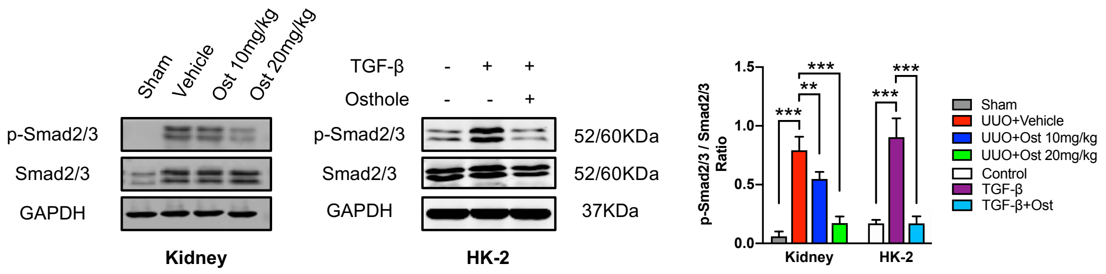

Supplementary Figure S1. Osthole inhibits the activation of Smad2/3 *in vivo* and *in vitro.* Representative western blots for p-Smad2/3 and Smad2/3 protein expressions in kidney and HK-2 cells; the quantification of p-Smad2/3 and Smad2/3 western blots. (n=6 mice/group or 4 repeats *in vitro*) All data are presented as means ± SD. *p <0.05, **p < 0.01, ***p < 0.001.
